# Early clinical management of autosomal recessive polycystic kidney disease

**DOI:** 10.1007/s00467-021-04970-8

**Published:** 2021-02-17

**Authors:** Max Christoph Liebau

**Affiliations:** Department of Pediatrics and Center for Molecular Medicine, Medical Faculty and University Hospital Cologne, University of Cologne, Kerpener Str. 62, 50937 Cologne, Germany

**Keywords:** *PKHD1*, Fibrocystin, Polycystic kidney disease, Ciliopathies, Perinatal kidney disease, Congenital hepatic fibrosis

## Abstract

Autosomal recessive polycystic kidney disease (ARPKD) is a rare but highly relevant disorder in pediatric nephrology. This genetic disease is mainly caused by variants in the *PKHD1* gene and is characterized by fibrocystic hepatorenal phenotypes with major clinical variability. ARPKD frequently presents perinatally, and the management of perinatal and early disease symptoms may be challenging. This review discusses aspects of early manifestations in ARPKD and its clincial management with a special focus on kidney disease.

## Introduction

Autosomal recessive polycystic kidney disease (ARPKD) is a rare disorder with an estimated incidence of 1 in 20,000 live births in Caucasians, corresponding to a carrier frequency of approximately 1:70 [1–6]. The disease still poses a major challenge in pediatric nephrology for patients, families, and caregivers. Over the last two decades, there has been considerable progress in our knowledge on this severe disorder, but many open questions remain. There is evidence for substantial, even intrafamilial, phenotypic variability between patients, but the most severely affected patients will present perinatally. This review therefore focuses on aspects of ARPKD management early in life.

## General clinical presentation and differential diagnoses

**Fig. 1:**
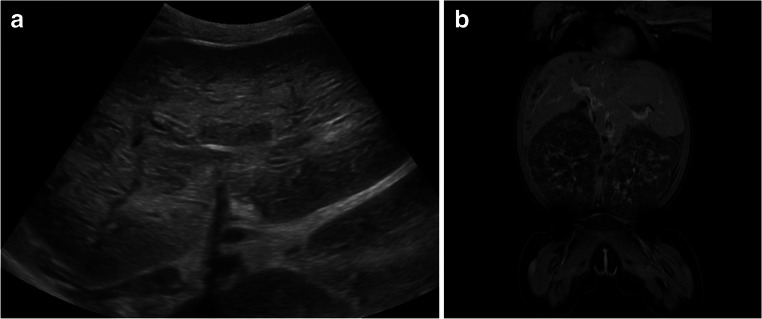
Radiological presentation of ARPKD with enlarged kidneys detected by ultrasound (**a**) and magnetic resonance imaging (**b**)

**Fig. 2:**
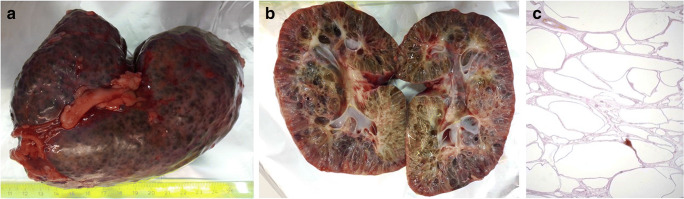
Pathologic presentation of ARPKD with reniform kidneys with ubiquitous cysts (**a**, **b**) and tubular dilatations in histology (**c**)

Clinically, the ARPKD kidney phenotype is typically characterized by bilateral massively enlarged reniform kidneys with poor corticomedullary differentiation on ultrasound (Fig. 1). Kidney enlargement is due to ubiquitous dilatations of the distal nephron, typically starting in the collecting duct [2]. This process frequently begins antenatally and results in the typical microcysts (Fig. 2). Macrocysts that may resemble an ADPKD-like phenotype may develop during the course of the disease, and the clinical differential diagnosis between ARPKD and very-early-onset forms of ADPKD (VEO ADPKD) may be difficult [7, 8]. Kidney function in ARPKD shows very variable courses ranging from antenatal impairment resulting in oligo- or anhydramnios to preservation of kidney function well into adulthood. It is estimated that about 50% of ARPKD patients will require kidney replacement therapy in the first two decades of life [5, 9–14]. It has also been estimated that up to 30% of the children may die shortly after birth from respiratory insufficiency, but it needs to be kept in mind that many of the estimations on survival come from times prior to recent developments in neonatal intensive care medicine and respiratory support. A more recent calculation for the USA revealed a survival rate of 79% for the period between 2010 and 2014 [6]. Furthermore, the 10-year survival of those patients being managed in the first month of life was previously found to be very high.

While the disease is called “autosomal recessive polycystic kidney disease,” a liver phenotype, which is initiated by a developmental defect of the bile ducts (ductal plate malformation), is obligatory in ARPKD. Interdisciplinary treatment should be established early in life. Liver disease can clinically present as congenital hepatic fibrosis (CHF) with variable bile duct dilatations of both intra- and extrahepatic bile ducts (Caroli’s syndrome) [15]. Over the course of the disease, this liver phenotype may result in portal hypertension with the risk of variceal bleeding, as well as in an increased risk of cholangitis. Cholangitis should actively be sought in an ARPKD patient with fever. Importantly, hepatocellular function typically remains stable. Thus, liver enzymes in serum in most cases are found in the normal range. Cholestasis parameters may be elevated [10, 14, 15]. ARPKD is one of the two major indications for pediatric combined liver and kidney transplantation, although clear-cut recommendations for this indication remain to be established [16–18]. There have been reports of both uniform and organ-specific disease progression in the liver and the kidney in ARPKD, and the underlying mechanisms remain incompletely understood [5, 9, 19]. In many ARPKD patients, liver disease clinically presents later than kidney disease [5, 20, 21] and thus is not the focus of this review. Important aspects of liver disease and its clinical management and follow-up are included in recent reviews [1, 3, 21] and the international consensus recommendations for diagnosis and management of ARPKD [10].

A clinical diagnosis of ARPKD can be made according to the modified Zerres criteria with typical kidney imaging findings and one or more additional criteria, which include typical liver imaging, typical clinical or laboratory signs of congenital hepatic fibrosis, liver pathology findings of biliary ductal plate malformation, absence of kidney enlargement in both parents or characteristic imaging findings in both parents as demonstrated by high-resolution ultrasonography, and pathology or genetic diagnosis of ARPKD in an affected sibling [3, 22]. Still, in modern times, offering postnatal genetic confirmation of the diagnosis has been recommended for early-onset bilateral cystic kidney diseases [7]. In addition to VEO ADPKD, the broad differential diagnoses include, e.g., Bardet-Biedl syndrome (BBS), *HNF1B*-nephropathy, cystic kidney dysplasia, infantile nephronophthisis, and metabolic disorders including defects of fatty acid oxidation [2, 7, 10].

## Genetics

ARPKD is an autosomal recessive disorder that affects males and females with equal incidences [5, 9, 13]. Siblings carry the risk of being heterozygous carriers. The disease is in most cases caused by variants in the gene *Polycystic Kidney and Hepatic Disease 1* (*PKHD1*) on chromosome 6 [23, 24]. Recently, variants in the gene *DAZ Interacting Zinc Finger Protein 1 Like* (*DZIP1L*) have been identified in a small group of patients with an ARPKD-like phenotype [25]. *PKHD1 e*ncodes a very large protein termed fibrocystin (also: polyductin) that contains a large extracellular part, a single transmembrane domain, and a short cytoplasmic tail [2, 23, 24]. Amongst other cellular sites, fibrocystin localizes to primary cilia and it has been suggested from preclinical and clinical data that the protein may be involved in the regulation of intracellular signaling pathways that are also affected in the more common autosomal dominant form of PKD (ADPKD) [26–30]. Yet, overall, fibrocystin’s function remains incompletely understood. A detailed description of the protein and its functions lies beyond the scope of this manuscript.

Given the mentioned differential diagnosis with partly overlapping phenotypes that may be clinically hard to differentiate from each other, the establishment of a genetic diagnosis in patients with an early-onset ARPKD phenotype may be helpful for counseling of families, e.g., on the risk of recurrence and, in some cases, on risks of disease progression [7, 10]. In addition, an established genetic diagnosis may point to extrarenal complications that may develop and may help in the development of a personalized medical management. This includes, e.g., a risk for autism spectrum disorders in patients with *HNF1B* deletions. A careful weighing between potential benefits and risks in the context of parents’ values and beliefs is required, and parents of young children need to be counseled in detail prior to performing genetic testing after informed consent. Where available, genetic testing or at least the information on genetic testing should, however, be offered to families [7].

Homozygous or compound heterozygous variants in *PKHD1* are found in approximately 80–90% of ARPKD patients [9, 12, 31–33]. At least one variant is found in up to 95% of families. The most frequent variant resulting in the T36M change of the protein affects approximately 10% of the screened alleles [34]. Genotype–phenotype correlations have remained loose, and interpretation of genotypes is challenged by the very large number of known private variants without a clearly defined hotspot and a complex *PKHD1* splicing pattern [34]. It is widely accepted that patients with two truncating variants show a more severe phenotype with more frequent peri- or neonatal demise. Milder phenotypes have been observed in patients carrying at least one missense variant. Yet, even the presence of two missense variants can go along with a severe phenotype that is not compatible with neonatal survival, and recent reports also show two truncating variants in some patients surviving the neonatal period [34–36]. In addition, intrafamilial variability and variability between liver and kidney phenotypes have been described [15, 37] and some patients with convincing *PKHD1* variants showed mild clinical effects even well into adulthood [20, 38]. Thus, utmost care is required when counseling families based on the genotype. More data is required. To get a deeper understanding of the clinical disease course, multiple initiatives have been initiated to gather longitudinal data [6, 39]. Such data may also become helpful for preimplantation genetic diagnosis, which is possible in ARPKD especially for expecting parents with a previous history of a child with severe disease course or couples with a high risk of recurrence. If the *PKHD1* variants have been identified in the parents, an embryo can be biopsied after in vitro fertilization for genetic testing [40, 41]. Non-invasive methods may become more relevant in the future. Medical, legal, and ethical aspects obviously need to be considered, including respect for autonomy and patient’s perspective as well as parents’ values and beliefs [42].

## Perinatal and early aspects of clinical presentation

In modern times, many ARPKD patients are identified antenatally. A typical phenotype includes enlarged and hyperechogenic kidneys with or without oligo- or anhydramnios although other differential diagnoses need to be considered with this presentation [7, 43, 44]. Oligo- or anhydramnios may result in the typical “Potter sequence” phenotype with pulmonary hypoplasia, a characteristic facies, and contracted limbs with club feet. While neonatal intensive care medicine has developed tremendously with sophisticated methods of mechanical ventilation and/or surfactant application, mortality remains substantial in severely affected ARPKD neonates. Prenatal lung assessment by ultrasound or MRI to predict postnatal course remains very challenging, but early detection of oligohydramnios seems to be associated with worse outcome. A recent recommendation found that there is insufficient data to support serial amnioninfusions [7]. The defined use of antenatal corticosteroids to support lung maturation in late preterms may be helpful. During the first days of life, the pulmonary situation is the crucial factor for the overall prognosis [8, 10]. Pneumothoraces may pose a problem. A description of the precise treatment of pulmonary hypoplasia is not within the focus of this review. General principles apply.

Given the potentially severe courses, prenatal detection of bilaterally enlarged and hyperechogenic kidneys with the suspicion of cystic kidney disease may lead to termination of pregnancy [45]. It is important to note that not all patients with antenatal proof of enlarged hyperechogenic kidneys suffer from ARPKD. As mentioned above, there are multiple differential diagnoses to be considered, including ADPKD, BBS, and *HNF1B* nephropathy. Given the nature of this situation, there is limited evidence to help individual counseling. A recent study of a large cohort found that postnatal kidney outcome was good in fetuses with isolated renal hyperechogenicity, with almost 80% having normal kidney function after birth. All children with echogenic kidneys and normal amniotic fluid levels had normal kidney function after birth. The presence of additional renal and extrarenal findings was important [44]. It is important to note that recent data suggest that *HNF1B* nephropathy shows good kidney survival [46]. In general, the prognosis of children with severely impaired kidney function from birth onwards is poorest in children with renal oligohydramnios but also depends on extrarenal comorbidities.

Prenatal tests of kidney function beyond the detection of oligo-/anhydramnios are not well established for cystic kidney diseases. Important general aspects of biochemical markers in fetal serum and urine have recently been reviewed [47]. A recent ARPKD-specific study from the ARegPKD consortium on 385 patients identified prenatal sonographic identification of enlarged kidneys, kidney cysts, and documentation of oligo-/anhydramnios as antenatal markers that may be helpful to predict early dialysis dependency in ARPKD patients [13]. The study compared 36 patients with the need for dialysis in the first year of life with 349 patients who did not require dialysis in this timeframe. Interestingly, the documentation of oligohydramnios or anhydramnios, prenatal kidney enlargement, a low Apgar score, and the need for postnatal breathing support showed an independent association with an increased hazard ratio for the need of dialysis within the first year of life according to multivariate Cox regression analysis. The antenatal isolated or combined detection of enlarged kidneys, kidney cysts, and documentation of oligo-/anhydramnios showed a gradual increase of probability of early postnatal dialysis dependency in a predictive model derived from the dataset [13]. More data will be required to validate and extend these markers. Recently, a proteomics-based approach has, e.g., been used to analyze amniotic fluid peptides as a marker to predict postnatal kidney survival in developmental kidney diseases, with a focus on the spectrum of congenital anomalies of the kidney and the urinary tract (CAKUT) [48]. Whether comparable approaches will have the potential to be applied to ARPKD remains to be determined.

Given the complexity of postnatal treatment of patients with suspected ARPKD, delivery in a hospital with specialized neonatal and pediatric nephrological care may be required [7, 10]. Severely affected children and their families will benefit from an interdisciplinary treatment. This includes situations in which the decisions for palliative treatment with restriction of intensive care including dialysis may have to be taken.

### Early kidney disease

As previously mentioned, kidney disease presentation in ARPKD can be very variable. Kidney failure is not a very common cause of neonatal demise, and kidney function may improve in the first months of life [49] as is also seen in other inborn kidney diseases of the CAKUT spectrum. In patients with a pronounced phenotype, urine output should be monitored from birth and serum creatinine and electrolyte levels as well as acid-base status should be monitored according to the clinical course. Overall, perinatally symptomatic ARPKD patients seem to have a worse long-term outcome of kidney function than children without perinatal presentation [11, 12]. For young children, management should, in principle, follow standard recommendations with peritoneal dialysis as the dialysis modality of choice [7, 10]. Overall, kidney replacement therapy in neonates and infants has been shown to result in about 80% survival after two years in a large international cohort [50]. In a cohort from North America, overall survival was about 80% after three years for children with initiation of kidney replacement therapy in the first month of life and 85% for children with initiation of kidney replacement therapy in the rest of the first year of life [51]. The situation may be more difficult in countries with limited resources. While early-onset kidney replacement therapy can be considered as an established treatment, the decision to start, to withhold, or to discontinue kidney replacement therapy may depend on multiple individual aspects of a patient and the family. Such a decision should be taken by the families and a multidisciplinary approach and may be supported by a formalized ethical decision-making framework [7, 10].

Concerns exist about the feasibility of peritoneal dialysis in ARPKD with enlarged kidneys. A recent study from the International Pediatric Peritoneal Dialysis Network (IPPN) registry therefore systematically compared peritoneal dialysis in children with ARPKD (*n* = 79) with control groups suffering from congenital nephrotic syndrome (*n* = 79) and CAKUT phenotypes (*n* = 158) [52]. Patients were matched for age and time on dialysis. As previously reported, CAKUT patients showed better overall survival than the two other groups, but there was no difference between ARPKD and CNS cohorts. There was no difference between the three groups in terms of technical peritoneal dialysis survival in a Kaplan-Meier analysis over several years. Peritoneal dialysis required minor adaptions of prescription in ARPKD. Interestingly, a higher ultrafiltration per glucose ratio was observed for some ARPKD patients, which may potentially be related to portal hypertension. It is important to note that the studied cohorts were cohorts of young children with a median baseline age of 2.4 years, but that the study was not designed to compare peritoneal dialysis in the first weeks of life. However, a sub-analysis of data from patients in the first year of life did not show differences between ARPKD patients and patients suffering from congenital nephrotic syndrome.

### Early nephrectomies in ARPKD and neurological sequelae

**Fig. 3:**
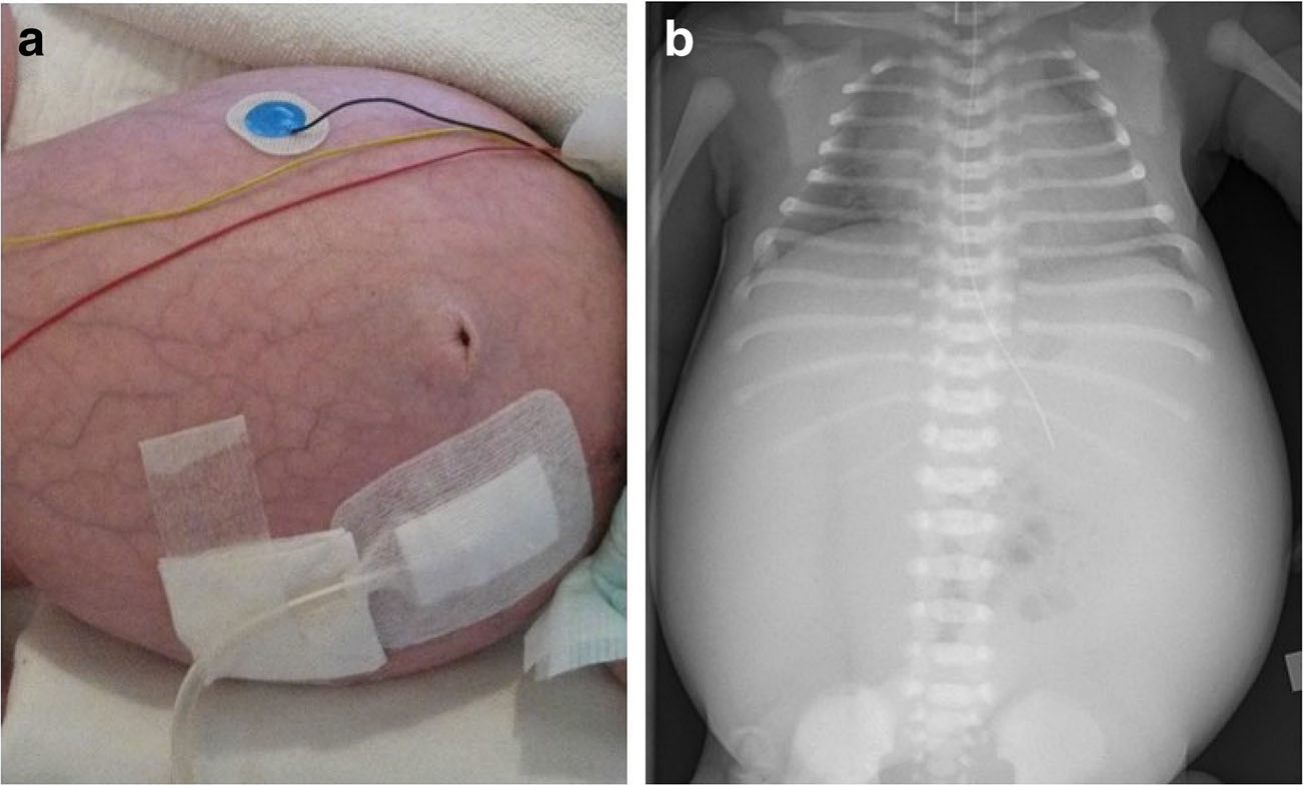
Clinical presentations of ARPKD with an enlarged abdomen (**a**) or pulmonary hypoplasia on chest radiography (**b**)

An important detail that could not be addressed by the IPPN study concerns nephrectomies for peritoneal dialysis in ARPKD. ARPKD may show massively enlarged kidney and pulmonary hypoplasia (Fig. 3), and uni- or bilateral nephrectomies have been suggested for pulmonary indications as well as to improve nutrition and blood pressure control [53–56]. Still, the evidence that supports potential benefits of nephrectomies is limited and needs to be weighed against the potential risks and sequelae of nephrectomies, e.g., resulting from loss of nephron mass and kidney function with a subsequent need for kidney replacement therapy [10]. In addition, there have been reports about arterial hypotension after bilateral nephrectomies that may result in neurological damage in young patients on peritoneal dialysis [57–60]. A recent ARegPKD study compared ARPKD children after very early bilateral nephrectomies (both nephrectomies within the first three months of life, VEBNE) with children after early bilateral nephrectomies (bilateral nephrectomies within the subsequent year), very early dialysis (start of dialysis in first three months of life), and a control group with large total kidney volume [61]. Severe neurological complications were most frequently documented in VEBNE patients in comparison to all control groups, with VEBNE and documented episodes of hypotension as independent risk factors for the observed severe neurological complications. Patients with early bilateral nephrectomies and very early dialysis showed more complications than the total kidney volume control group. These data may support a very cautious approach towards bilateral nephrectomies, especially in the first months of life during which, e.g., maturation processes of autonomic cardiovascular control still seem to take place [61]. Rapid growth of the remaining kidney after unilateral nephrectomy has been observed in some cases requiring a second nephrectomy after a short time interval after the first nephrectomy. It should therefore be carefully considered whether some time can be gained prior to a first or a second nephrectomy that may be inevitable.

### Hypertension and hyponatremia

A common and severe problem of ARPKD in the first few months of life is arterial hypertension that may be very severe and may require treatment with multiple drug classes. Inhibitors of the renin-angiotensin system are considered to be the treatment of choice due to experimental observations and general considerations [62–64]. The underlying pathophysiology resulting in the partly massive hypertension in ARPKD is not well understood, but dysregulation of renal sodium and volume handling with impaired urinary dilution and an activation of the renin-angiotensin system have been suggested. It has been suggested that hypertension may improve as kidney function declines. Hypertension may be aggravated by substitution of sodium chloride given for hyponatremia that is frequently observed as a transient finding in ARPKD mainly in the first year of life. For treatment of hyponatremia in ARPKD, general principles apply. In euvolemic or hypervolemic patients, fluid intake with nutrition should be minimized, e.g., by concentrating feeds [10]. Low normal sodium levels may need to be tolerated.

## Early extrarenal aspects

Failure to thrive is a general concern in children with early-onset CKD but may pose a specific challenge in children with ARPKD. Enlarged kidneys and respiratory challenges of pulmonary hypoplasia in addition to uremia may affect feeding and nutrition early in life. Peritoneal dialysis will require filling of the abdominal cavity, which may add up with enlarged kidneys, although the previously mentioned study on peritoneal dialysis in ARPKD did not find differences in growth or body mass index between the three groups that were compared. The importance of gastrostomies for long-term supplemental or exclusive enteral tube feeding in children with CKD has recently been pointed out by the pediatric renal nutrition taskforce [65]. There have, however, been concerns about gastrostomies in ARPKD as there have been reports on an increased risk of spleen injury in cases of splenomegaly in patients with liver disease and portal hypertension due to other underlying disorders. Furthermore, the presence of stomal varices due to portal hypertension could be a concern. Such considerations resulted in the classification of ARPKD as a relative contraindication for gastrostomy insertion [66, 67].

A recent survey therefore questioned pediatric nephrology and pediatric gastroenterology/hepatology centers on their opinions and experiences with gastrostomy insertion in ARPKD patients [67]. There were almost 200 participants from 39 countries with most of them from pediatric nephrology centers. Real-life data was retrospectively assessed for benefits and complications in a group of 38 ARPKD patients. Most of the participants in principle supported gastrostomy tube insertion for ARPKD patients if required, e.g., due to insufficient oral intake of calories. Reported complications in ARPKD patients included were in principle comparable to those of non-ARPKD patients, but the limitations of the survey approach need to be kept in mind. More in-depth clinical data would be required as a foundation for clinical recommendation. Still, a large majority of the centers contributing data on patient courses retrospectively considered that the gastrostomy approach was the right decision for their patients. For daily clinical life, it may be wise to approach and team up with the colleagues and centers placing gastrostomy tubes (e.g., pediatric gastroenterologists, pediatric hepatologists, pediatric surgeons, or a corresponding experienced referral center) very early during the course of the disease.

In addition to this survey, an analysis of the CKiD cohort analyzed growth in a group of ARPKD patients with a mean age of 7.9 years and compared findings to two matched control groups with other congenital causes of chronic kidney disease. In this older-age group, there was no evidence for a disease-specific effect of ARPKD on growth [68].

It is obvious that families of severely affected children will greatly benefit from specific nutritional consulting. Furthermore, the value of psychosocial support can hardly be overestimated for families of children with such an early-onset chronic kidney disease. This includes contact with a patient advisory group like PKD International. A detailed description of the importance and the benefits of psychosocial support and connections for families lies beyond the scope of this manuscript.

## Outlook and summary

Autosomal recessive polycystic kidney disease remains a challenge in pediatric nephrology. A lot has been learned over the past two decades, but multiple open questions remain. There is still an urgent need to improve our understanding of the disease course and to identify additional specific, early, and precise prognostic risk markers for kidney and liver disease, as well as for prenatal prediction of the postnatal respiratory situation. Such markers would help to identify patients who might either benefit from intense treatment approaches or not even require such interventions. Furthermore, our understanding of the molecular mechanisms resulting in this severe disease remains by far too limited and much more work will be required as a basis for the development of novel and disease-specific therapeutic approaches. The impressive development in the field of ADPKD may serve as a template for some of the upcoming steps, but additional, ARPKD-specific issues require to be taken into account, again including pre- and perinatal aspects.

### Key summary points

- Management of early autosomal recessive polycystic kidney disease (ARPKD) remains a challenge in pediatric nephrology.
- ARPKD can present antenatally with hyperechogenic and enlarged kidneys, but differential diagnoses of such ultrasound findings need to be considered.
- The extent of pulmonary hypoplasia after renal oligo-/anhydramnios is crucial for prognosis in the first days of life. Very early bilateral nephrectomies may be associated with poor neurological outcome.
- Kidney replacement therapy is required in about 50% of ARPKD patients in the first two decades of life. Peritoneal dialysis has been recommended as the dialysis modality of choice early in life.

### Multiple choice question

1. Which of the following statements is true?
  - ARPKD is mainly caused by variants in the *PKHD1* gene.
  - Variants in *DZIP1L* are more common than variants in *PKHD1.*
  - Specific variants in *DZIP1L* are always associated with specific variants in *PKHD1.*
  - *PKHD1* encodes polycystin-1.
  - *DZIP1L* encodes the ARPKD protein fibrocystin.
2. Which of the following statements is true?
  - With the knowledge of the genotype, clear-cut prediction of the clinical course of an ARPKD patient is possible.
  - Two missense variants in *PKHD1* always result in a mild phenotype.
  - Two truncating variants in *PKHD1* typically result in a severe phenotype that may not be compatible with life.
  - A missense and a null variant in *PKHD1* result in an intermediate phenotype.
  - With the knowledge of the clinical course of a sibling, clear-cut prediction of the clinical course of an ARPKD patient is possible.
3. Which of the following statements is true?
  - In ARPKD kidney function determines survival in the first days of life.
  - Liver disease typically shows the earliest symptoms compared to other affected organs.
  - Kidney disease in ARPKD results in a need of kidney replacement therapy in the first year of life in 80% of affected children.
  - Patients with prenatal manifestation and oligohydramnios cannot survive.
  - Kidney enlargement, kidney cysts, and oligo-/anhydramnios may be helpful to estimate a risk for early postnatal dialysis dependency.
4. Which of the following statements is true?
  - Peritoneal dialysis is not possible in ARPKD.
  - Peritoneal dialysis is not possible in ARPKD without obligatory nephrectomies.
  - Peritoneal dialysis should only be offered to ARPKD children without liver disease.
  - Peritoneal dialysis can be applied in ARPKD but may require adaptations.
  - Peritoneal dialysis is not needed in ARPKD as kidney function can be preserved through targeted pharmacological intervention.
5. Which of the following statements is true?
  - Hypertension is not an issue in ARPKD.
  - Hypertension can be very pronounced in ARPKD requiring treatment with multiple pharmacological classes.
  - Hypertension is only an issue in adult patients with ARPKD.
  - Hypertension in ARPKD should be treated by nephrectomy.
  - Hypertension in ARPKD results in obligatory variceal bleeding.
